# The Impact of COVID-19 on Families With Pediatric Muscular Dystrophy Patients

**DOI:** 10.7759/cureus.41138

**Published:** 2023-06-29

**Authors:** Adeel S Zubair, Kirsten Scharer, Paige Lembeck, Cristian Ionita, Bhaskar Roy

**Affiliations:** 1 Neurology, Yale School of Medicine, New Haven, USA; 2 Psychiatry, Children’s Hospital of Philadelphia, Philadelphia, USA

**Keywords:** psychological impact, neuromuscular, muscular dystrophy, pediatrics, covid-19

## Abstract

The coronavirus disease 2019 (COVID-19) pandemic resulted in unprecedented changes in daily activities and healthcare services. In the United States, stay-at-home orders and social distancing measures were put, and school closures impacted many students. The psychological impact of the COVID-19 pandemic has been shown to have wide-ranging and long-term effects. With school closures and limitations in in-person visits and provider care, we hypothesized that the patients with pediatric muscular dystrophies and neuromuscular conditions were more vulnerable to the restriction posed by this pandemic. This survey-based study examined the psychosocial impact of this pandemic on pediatric patients with neuromuscular disorders and caregiver burden through chart review and self-reports via survey administration using a validated tool (COVID-19 Exposure and Family Impact Scales {CEFIS}). The majority of families reported that they had a stay-at-home order (91.7%), schools/childcare centers were closed (87.5%), their children’s education was disrupted (83.3%), and they were unable to visit or care for a family member (58.3%). Parents/caregivers felt that the COVID-19 pandemic made parenting a little bit worse (mean = 2.6 ± 0.96) and made it more difficult to care for the elderly or those with disabilities in the family (mean = 2.6 ± 0.95) and for their child with a neuromuscular disability (mean = 2.6 ± 0.91). Our data highlights the significant impact of the COVID-19 pandemic on the lives of families and caregivers of pediatric patients with muscular dystrophies.

## Introduction

The coronavirus disease 2019 (COVID-19) pandemic resulted in unprecedented changes in daily activities and healthcare services. In the United States, stay-at-home orders and social distancing measures were put into place starting in March of 2020, and schools were closed impacting about 55 million students [1,2]. Such measures were necessary to prevent the uncontrolled spread of the virus but affected both children and their families [3].

The psychological impact of the COVID-19 pandemic has been shown to have wide-ranging and long-term effects [4,5]. During the pandemic, conflicts at home have increased for all family members including children, as has anxiety and depression [6,7]. The pandemic has been a major burden on families; however, in particular subgroups, this burden has been amplified. These groups include the parents of children with physical or mental health conditions. Previous studies have shown that these groups of parents report more parental burnout and have less perceived social support [2-4].

Pediatric muscular dystrophies are rare inherited disorders affecting 3.8-26.8 individuals per 100,000; however, they often have significant disease burden and require multidisciplinary management [8]. These conditions are progressive in nature and require monitoring and intervention from physical therapy, social workers, occupational therapy, and speech-language pathology. Many schools assist with the delivery of these resources to the patients. With school closures and limitations to in-person visits and provider care, we hypothesized that this subset of patients was susceptible to the restrictions posed by this pandemic.

To the best of our knowledge, no studies have investigated the levels of caregiver burden and stress in families and pediatric patients with neuromuscular disorders during the COVID-19 pandemic. This study examined the psychosocial impact of this pandemic on pediatric patients with neuromuscular disorders and caregiver burden through chart review and self-reports via a validated survey administration.

## Materials and methods

This study was approved by the Yale Institutional Review Board (IRB). The patients currently being followed in the Yale Pediatric Muscular Dystrophy Clinic, who have a neuromuscular disorder, were identified through a medical record search. After the patients were identified, a member of the study team went through their electronic medical records to ensure that all patients met the eligibility criteria, which included having a muscular dystrophy/neuromuscular disorder and an age under 18 and being seen at least once in the Pediatric Muscular Dystrophy Clinic. Overall, 187 patients were identified who met the eligibility criteria (Table 1). Of this list, caregiver email information was available for 145. All parents/caregivers of this group of patients were contacted with a link to the survey, and we received responses from 24 caregivers.

**Table 1:** Demographic table of the overall cohort

Number of patients	187
Average age (years)	11.19
Female (percentage)	35.30%
Diagnoses	
Myotonic dystrophy	9.60%
Muscular dystrophy	41.20%
Congenital myopathy	9.60%
Hereditary spastic paraplegia	2.10%
Myasthenia gravis	6.90%
Disorder of metabolism	3.20%
Spinal muscular atrophy	8.00%
Ataxia syndromes	3.70%
Others	15.50%

The survey used in the study was the COVID-19 Exposure and Family Impact Scales (CEFIS). The COVID-19 Exposure and Family Impact Scales (CEFIS) are based upon a trauma framework (Substance Abuse and Mental Health Services Administration, 2014) and were created to understand how COVID-19 affects families [9]. This measure was validated through a multidisciplinary and multi-institutional team and has been used in studying caregiver impact in other pediatric populations. The first part of the survey consisted of “yes” or “no” questions that inquired about the occurrence of COVID-19-specific events. The second part of the survey consisted of items assessing the impact of COVID-19 on various domains in life (e.g., parenting), with response options on a Likert scale of 1-4, with 1 being “made it a lot better” and 4 being “made it a lot worse” [9]. The CEFIS is included in Appendices. Descriptive statistics were represented as mean ± standard deviation (SD) if not otherwise specified.

All study responses were stored in Qualtrics (Qualtrics International Inc., Seattle, WA). All identified families were contacted a maximum of three times via email. Two members (KS and AZ) of the study team independently evaluated and coded text responses to open-ended survey questions using thematic analysis [10]. These themes were derived from the participants’ own words rather than imposing an existing framework [11]. Consensus on a final list of codes was reached using an iterative process of discussion and comparing codes. The coded responses were grouped into higher-level themes [10].

## Results

We attempted to contact 145 parents/caregivers and obtained completed results from 24 (16.6%). The majority of families reported that they had a stay-at-home order (91.7%), schools/childcare centers were closed (87.5%), their children’s education was disrupted (83.3%), and they were unable to visit or care for a family member (58.3%) (Table 1). Of all the families, 66.7% reported that they had an essential worker in their household who had to continue to work during the pandemic. A minority of families had difficulty getting food (4.1%) or getting medicine (4.1%). A larger group had difficulty accessing medical care (16.7%). The pandemic did result in the loss of income (29.2%) and the loss of work hours (29.2%) with 16.7% of respondent families having a member permanently lose their job. The vast majority of families (83.3%) missed out on an important family event due to the pandemic. Of our cohort, 20.8% of families had a member diagnosed with or experiencing symptoms of COVID-19 as of June 2021.

Descriptive analyses revealed that on average, parents/caregivers felt that the COVID-19 pandemic made parenting a little bit worse (mean = 2.6 ± 0.96) and made it more difficult to care for the elderly or those with disabilities in the family (mean = 2.6 ± 0.95) and for their child with a neuromuscular disability (mean = 2.6 ± 0.91). Caregivers noted detriment in their sleep (mean = 2.9 ± 0.87) and their mood (mean = 3 ± 0.86) and worsened anxiety (mean = 3.2 ± 0.87).

Qualitative analysis

The responses from survey participants fell into three main themes: social impact, academic impact, and stress on caregivers and children. Social impact on both caregivers and children was reported to be both positive and negative. The participants expressed being grateful for spending more time together as a family; however, they also acknowledged social isolation.

Children missed activity and socialization with peers (Participant #10).

My stepdaughter lost some friends because she couldn’t go to school or see them (Participant #24).

The academic and therapeutic impact of COVID-19 was noted to be significant by those families whose children receive support and therapy from school. Families had to adapt to remote learning and technology.

We spent hours every night trying to complete work, and the teachers were completely unprepared for distance learning and constantly making mistakes with technology (Participant #7).

Kids suffered in school especially our son with special needs. He basically has lost a year of therapies. We had to hire a tutor just to try to help him (Participant #5).

Stress due to COVID-19 had a significant impact on caregivers, as well as children. Stress came from uncertainty, difficulty attaining consistent medical care, and anxiety about becoming ill with the virus. Caregivers faced an additional burden of providing academic and therapeutic support for their children.

In the beginning, in the spring of 2020, it was very difficult to help my daughter during distance learning. Because she is special needs, trying to navigate the classes with her and having no extra help was very difficult (Participant #7).

I have two special needs kids, one who’s severely medically fragile and couldn’t leave the house or we couldn’t let anyone visit. My son with ASD, ADHD, and anxiety disorder had to do distance learning all year to keep his sister safe and really struggled because he has social issues to begin with (Participant #21).

## Discussion

Our data highlights the significant impact of the COVID-19 pandemic on the lives of families and caregivers of pediatric patients with muscular dystrophies. There was increased caregiver stress, as well as social and academic impact on the patients. Patients with muscular dystrophy and other neuromuscular conditions have significant needs from behavioral, therapy, and treatment perspectives and require a multidisciplinary team to deliver care. The COVID-19 pandemic and disruptions to schooling increased the difficulty in providing care to these patients, especially since many are receiving rehabilitation therapies at school as part of special education services. Other commonly seen disruptions included limited interaction with the outside world and difficulty with access to healthcare.

The overall psychosocial impact of COVID-19 on children and parents has received limited attention [12]. A Canadian study showed that 70.2% of children and adolescents performed worse in at least one mental health domain compared to pre-pandemic, with social isolation being deemed a significant risk factor [13]. Other studies showed that the pandemic resulted in pediatric patients increasing their screen time and having less physical activity and parents reporting changes in children’s behavior and emotional state [6]. Studies on pediatric patients with chronic diseases are even fewer but showed stress, anxiety, depression, and insomnia among parents with pediatric kidney disease [14], celiac disease [15], and type 1 diabetes [16]. Our data also highlights that the impact on the patients and their families included most facets of their lives. Roughly a third of caregivers (29.2%) reported the loss of income secondary to the pandemic. There was a lack of social activity, and many could not attend an important family event. This theme of the loss of social interaction was also evident in the qualitative part of the study. Furthermore, negative impacts on mood, sleep, and anxiety were among the highest areas affected by COVID-19 based on average quantitative responses. However, in the absence of a control group with healthy children, a direct comparison with the general population was not possible, and the use of different types of surveys and scales between studies makes it hard to directly compare our study with others. As a result, we cannot comment on whether this particular cohort was more vulnerable to the COVID-19 pandemic compared to their healthy peers or to other children with chronic illness.

The stress on caregivers was also evident in the qualitative answers. The stress they reported originated from uncertainty, difficulty achieving consistent medical care, and anxiety about becoming ill (including with COVID-19). Caregivers had an additional layer of reported burden in providing academic and therapeutic support for their children.

This study only provides a cross-sectional analysis of the problem; the true impact of the pandemic on this population will only be apparent with longer-duration follow-up studies. The possible limitation of physical and mental activities during this period may have a long-term significant impact on this population.

This study has several limitations, and some of them are inherent to a pilot survey-based study. We did not have a control group with healthy children to compare the responses. The response rate was low, and the question set was limited. Given the anonymous nature of the survey, we could not ascertain the subgroup of ages and neuromuscular disorders covered by the survey responders. In the absence of such demographic data, the exact type of neuromuscular disorder and the baseline severity of their disease could not be confidently ascertained. However, as the survey population came only from the Yale Pediatric Muscular Dystrophy Clinic, we can be confident about their diagnosis of an underlying neuromuscular disorder, even in the absence of individual information. Nevertheless, the lack of precise demographic data and the small sample size limit the generalizability of these findings. However, pediatric neuromuscular disorders are rare, and a small sample size is not unusual for this population [17-24]. Other limitations include possible selection bias, in particular sampling and volunteer bias subtypes. However, we have sent reminder emails (a maximum total number of three emails) when there was no immediate response, to limit sampling bias, and the anonymous collection of responses should have addressed potential volunteer bias to some extent. In conjunction with the assessment of caregiver burden, a perspective from the affected patients themselves using self-report will provide further valuable information and is an area for future research. Additionally, future studies can also focus on neurologist and neurology nursing perspectives, as well as physical medicine and rehabilitation/psychiatry perspectives.

With those limitations in mind, this study serves as a basis for larger studies to tackle this important area in order to improve care delivery systems and help relieve caregiver burden. The results support the hypothesis that many families reported experiencing negative events due to COVID-19.

## Conclusions

The COVID-19 pandemic has caused a significant impact on the entire world; the patients and caregivers of patients with pediatric neuromuscular disorders were also affected. This study illustrated the psychosocial impact of this pandemic on pediatric patients with neuromuscular disorders. Patients were affected socially and academically, and there was also an increased caregiver burden. Future studies are warranted to develop innovative ways to mitigate the caregiver burden on this population and to ensure continuous quality care for these patients.

## Figures and Tables

**Figure 1:**
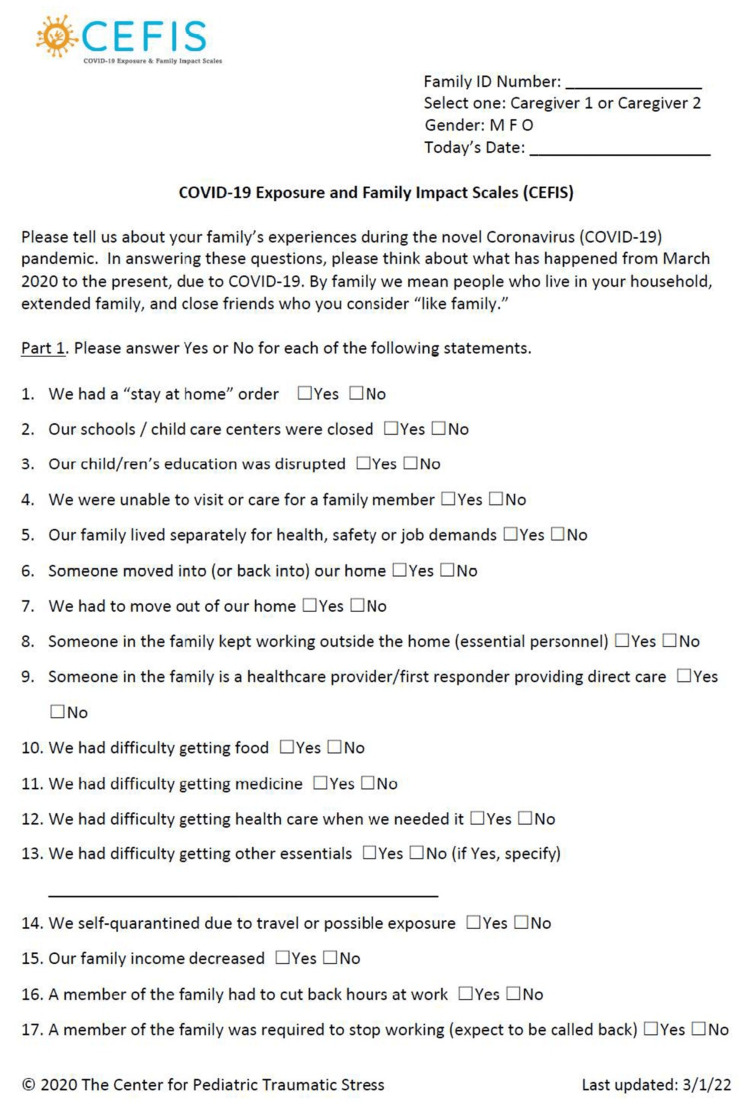
CEFIS survey page 1 COVID-19: coronavirus disease 2019

**Figure 2:**
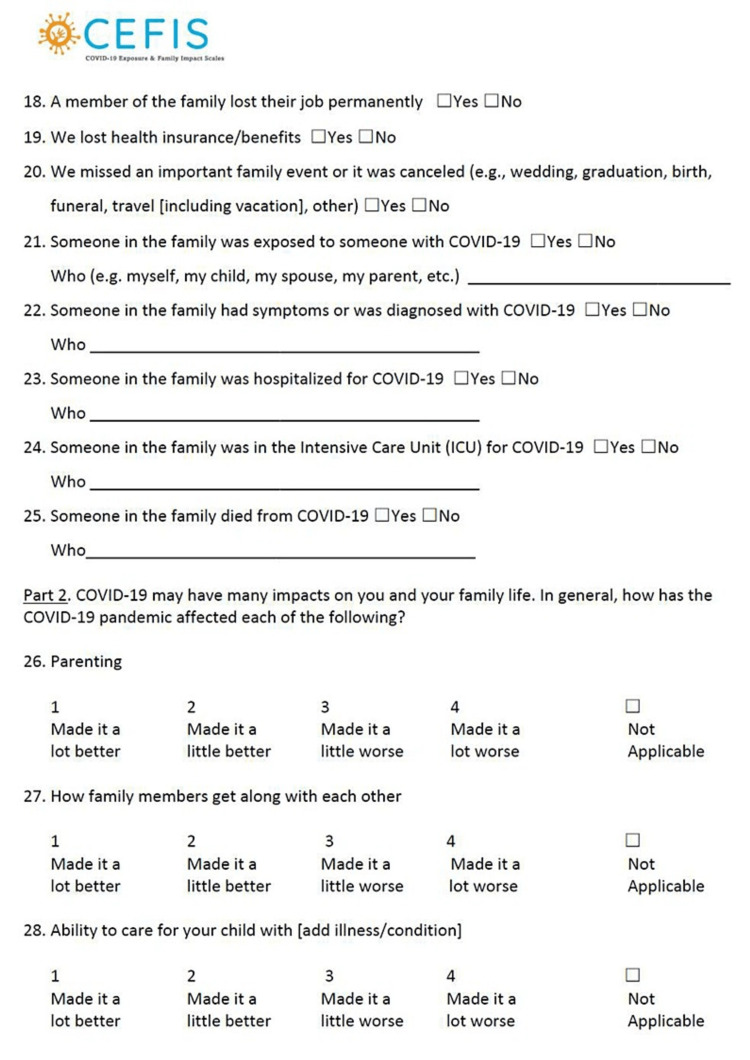
CEFIS survey page 2 COVID-19: coronavirus disease 2019

**Figure 3:**
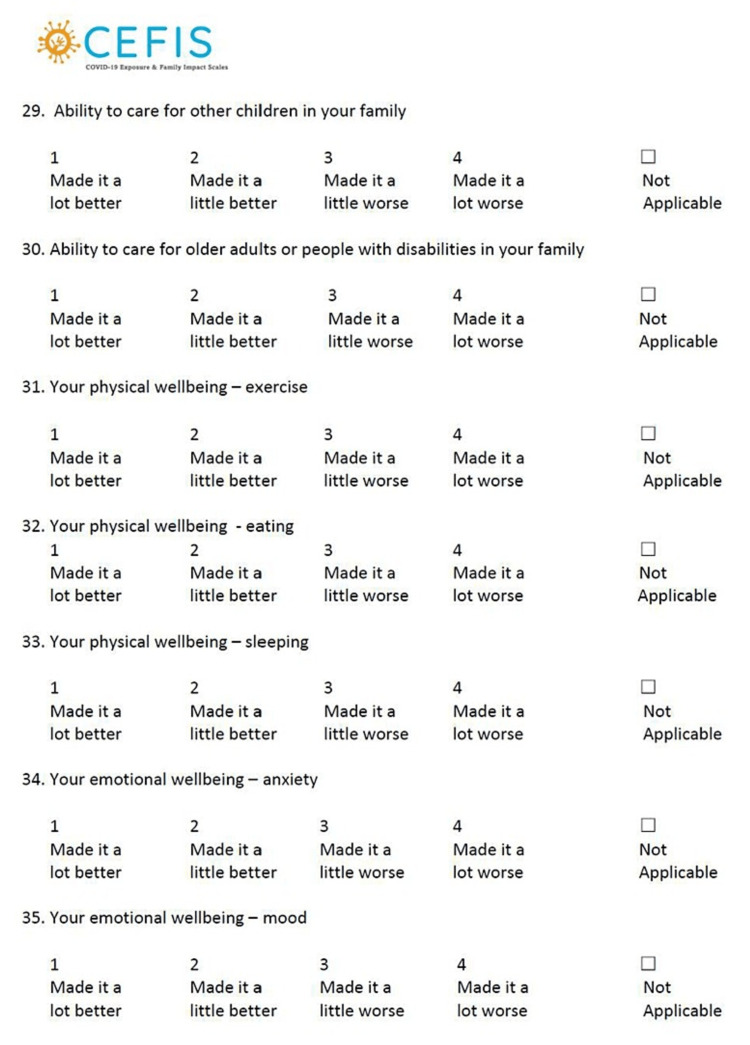
CEFIS survey page 3

**Figure 4:**
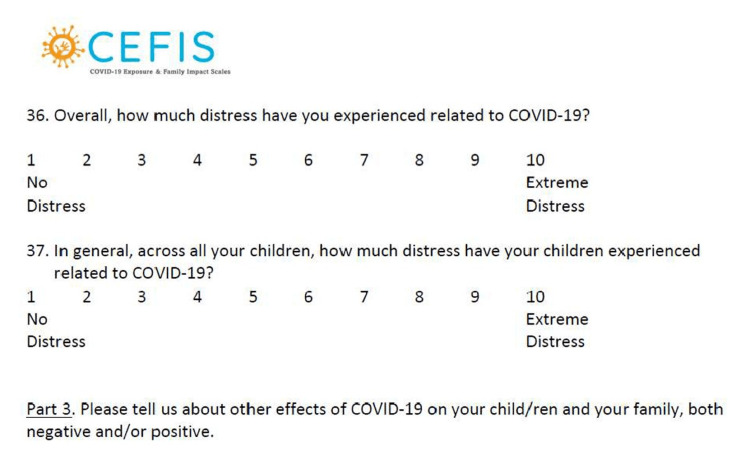
CEFIS survey page 4 COVID-19: coronavirus disease 2019

## Appendices

The CEFIS survey is shown in Figures 1-4. 
